# Possibility of CO_2_ laser-pumped multi-millijoule-level ultrafast pulse terahertz sources

**DOI:** 10.1038/s41598-023-51139-4

**Published:** 2024-01-10

**Authors:** György Tóth, Gergő Illés, Gabit Nazymbekov, Nelson Mbithi, Gábor Almási, János Hebling

**Affiliations:** 1 Institute of Physics, University of Pécs, Pecs, 7624 Hungary; 2 Szentágothai Research Centre, University of Pécs, 7624 Pecs, Hungary; 3 Natural Sciences Department, Garissa University, Garissa, 1801 Kenya; 4 HUN-REN-PTE High-Field Terahertz Research Group, Pecs, Hungary

**Keywords:** High-field lasers, Ultrafast lasers

## Abstract

In the last decade, intense research has been witnessed on developing compact, terahertz (THz) driven electron accelerators, producing electrons with a sub-MeV—few tens of MeV energy. Such economical devices could be used in scientific and material research and medical treatments. However, until now, the extremely high-energy THz pulses needed by the THz counterparts of the microwave accelerators were generated by optical rectification (OR) of ultrafast Ti:sapphire or Yb laser pulses. These lasers, however, are not very effective. Because of this, we use numerical simulations to investigate the possibility of generating high-energy THz pulses by the OR of pulses produced by CO_2_ lasers, which can have high plug-in efficiency. The results obtained supposing optical rectification (OR) in GaAs demonstrate that consideration of the self-phase-modulation (SPM) and the second-harmonic-generation (SHG) processes is indispensable in the design of CO_2_ laser-based THz sources. More interestingly, although these two processes hinder achieving high laser-to-THz conversion efficiency, they can still surpass the 1.5% value, ensuring high system efficiency and making the CO_2_ laser OR system a promising THz source. Our finding also has important implications for other middle-infrared laser-pumped OR-based THz sources.

## Introduction

Optical rectification of pulse front tilted ultrashort laser pulse in lithium niobate (LN) crystal is one of the most effective methods to generate THz pulses with either medium or high pulse energy and high peak electric field^1–3^. Medium energy THz pulses with μJ level energy and hundreds of kV/cm field generated by this method are suitable for and widely used in nonlinear THz spectroscopy and material control experiments^4,5^. Furthermore, they are used in electron acceleration^6,7^ and proposed for proton acceleration^8^. These later applications would enormously benefit from higher available THz energy.

However, using LN crystal, the large (~ 63°) needed intensity front tilt means a strong hindrance to significantly increase the THz energy further^9–11^. Some new techniques^12–14^, based on micro-machined crystals, can reduce the aforementioned limiting factors. However, manufacturing these crystals is immature and did not provide significant breakthroughs today^15,16^.

Semiconductor crystals are also applicable for generating good quality THz pulses with high efficiency. However, the smaller bandgap requires a long pumping wavelength to eliminate low-order multiphoton absorption^17–19^. With long wavelength pumping, the phase matching condition is no longer met in a co-linear case, so pulse front tilting must be used^17^ similarly to LN. The longer pumping wavelength and smaller (< 30°) needed tilt angle make it easier to adopt the new techniques, and by that, we can get a well-scaling THz source that operates with excellent efficiency^20^. However, generating the typically needed 1.7–4.0 μm wavelength^21^ pump pulses is usually done in an optical parametric amplifier (OPA), which has a decreasing efficiency for long wavelengths, resulting in low system efficiency. An efficient pump laser with the desired wavelength without wavelength conversion would be a solution. Carbon dioxide (CO_2_) lasers are known for their large plug-in efficiency^22^, and very recently, the production of only 2.0 ps long pulses with as large as 10 J energy at around 10 μm has been demonstrated ^23^. Furthermore, the numerical simulation predicts that in the presence of anomalous dispersion caused by selecting an appropriate isotope of CO_2_ gas, self-compression of a few ps duration pulses shorter than 0.5 ps is possible in CO_2_-filled cells^24^. Besides this, conventional multipass cell^25–27^ or hollow core fiber^28–30^ based techniques can be suitable to produce a modest, one-order of-magnitude shortening of CO_2_ laser pulses having less energy (< 1 J).

This manuscript will show that CO_2_ lasers are potentially suitable for pumping semiconductor crystals to generate THz pulses efficiently. The pump pulse length is assumed to be in the range of 0.5–2.5 ps.

As a typical nonlinear crystal in the middle-infrared range, we consider GaAs, an isotropic semiconductor having a zinc-blende structure with a symmetry class of $\overline{4}$3. It has a direct bandgap of 1.43 eV, a refractive index of about 3.6 in the optical region, a large nonlinear coefficient, $d_{14} = 134\;\;{\text{pm/V}}$, at 10.6 μm^31^, and a broad transmission range from 0.97 to 17 μm^31^. The damage threshold of 2 GW/cm^2^ was observed in GaAs at a pulse length of 250 ps^32^ and wavelength of 10 μm. According to the scaling law^33^, the damage threshold in the case of 2.5 ps could be 100 GW/cm^2^ and higher for a shorter pulse duration.

Since it is known that second harmonic generation (SHG) of CO_2_ laser pulses in GaAs wafer-stack is possible with as high as 2% conversion efficiency, and the SHG coherence length is larger than 100 μm^34^, in the numeric calculations, besides of the OR, the SHG was also taken into account. The numeric model also considers the absorption of the THz signal, and dispersion of both the pump and the THz signal, as well as the self-phase modulation of the pump.

## Methods

**Figure 1:**
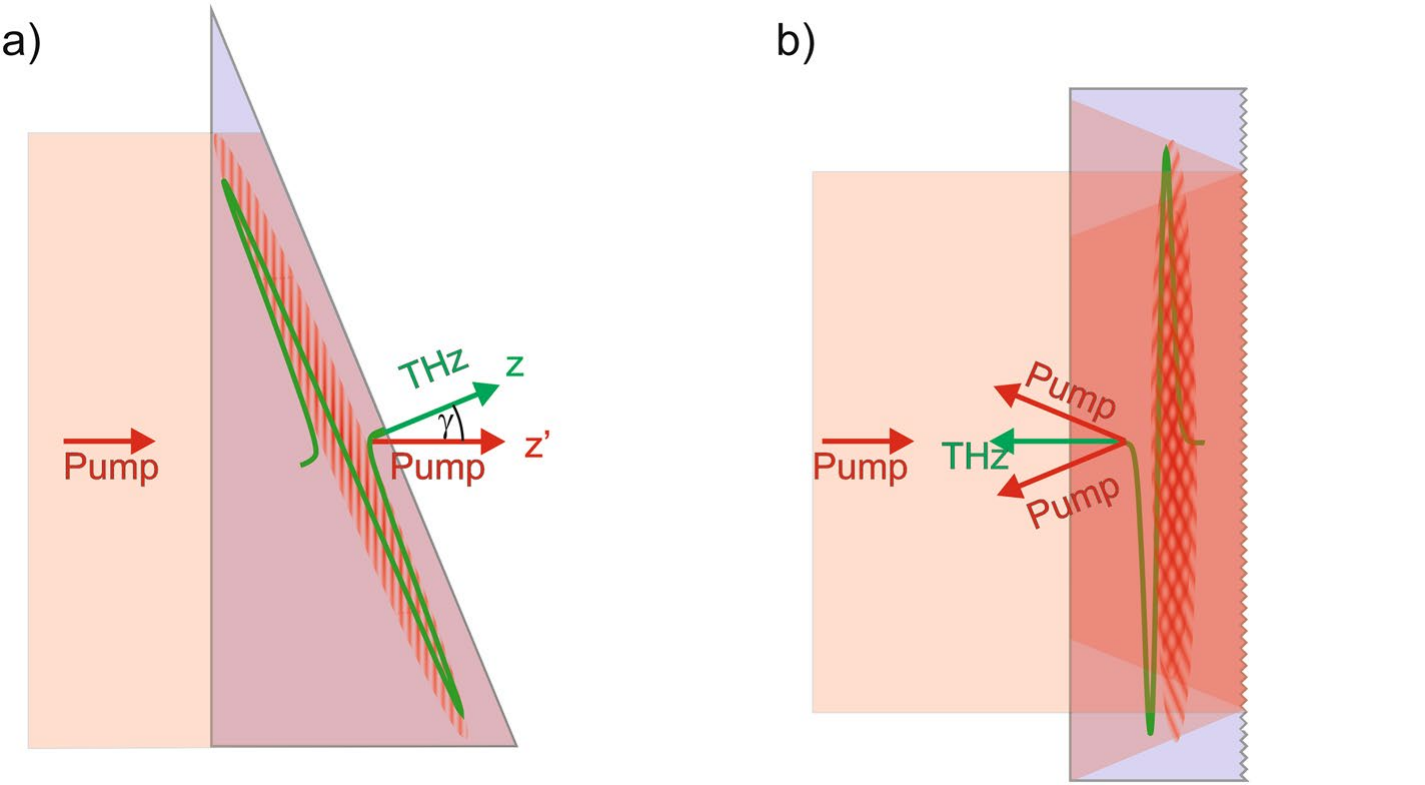
(**a**) Schematic drawing of a conventional TPFP: The pump pulse with a tilted intensity front enters the crystal from the left and propagates in the z′ direction. During its propagation, THz radiation is generated along the tilted intensity front and propagates in the z-direction. (**b**): New generation THz source: A non-tilted pulse front pump enters the crystal from the left and propagates towards the structured back surface of the plane-parallel crystal. Diffraction on the structured back surface causes tilting of the pulse intensity front of the backward propagating pump pulse. The generated THz pulse propagates opposite to the original propagation direction of the pump pulse.

Figure 1 shows schematically the propagation directions of the pump and THz pulses, as well as the pump intensity front in the nonlinear optical crystal of a conventional^35^ (a) and a new generation^36^ (b) THz pulse source using the tilted-pulse-front-pumping (TPFP) technique to achieve velocity matching between the pump and THz pulses. The red lines represent the phase fronts of the pump beam propagating along the z' direction. The pulse front of the pump beam (illustrated by shaded area) is tilted compared to the phase front by an angle of γ. The THz beam is generated perpendicularly to the pulse front and propagates in the z-direction.

Besides the OR, the following effects are taken into account in the numerical calculation of the THz generation process: pump pulse changes due to the linear dispersion, angular dispersion caused by the pulse front tilting, cascaded up- and down-conversion, self-phase modulation (SPM), and second harmonic generation (SHG). These effects dramatically influence the pump pulse and, consequently, the THz generation process. A 1 + 1D THz generation model is used in the calculations^9,37^.

The following differential-equation system was used to describe the processes mentioned above and solved numerically with the 4th-order Runge-Cutta method:

$$\begin{aligned} \frac{{dA_{THz} \left( {\Omega ,z} \right)}}{dz} & = - \frac{\alpha \left( \Omega \right)}{2}A_{THz} \left( {\Omega ,z} \right) \\ & \;\; - i\frac{{\Omega \chi_{eff}^{\left( 2 \right)} }}{{2cn_{THz} \left( \Omega \right)}}\mathop \smallint \limits_{0}^{\infty } A_{p} \left( {\omega + \Omega ,z} \right)A_{p}^{*} \left( {\omega ,z} \right)e^{{ - i\left( {\left( {k_{p} \left( {\omega + \Omega } \right) - k_{p} \left( \omega \right)} \right)/cos\left( \gamma \right) - k_{THz} \left( \Omega \right)} \right)z}} d\omega \\ \end{aligned}$$

$$\begin{aligned} \frac{{dA_{p} \left( {\omega ,z} \right)}}{dz} & = - i\frac{{\omega \chi_{eff}^{\left( 2 \right)} }}{{2cn_{p} \left( \omega \right)}}\mathop \smallint \limits_{0}^{\infty } A_{p} \left( {\omega + \Omega ,z} \right)A_{THz}^{*} \left( {\Omega ,z} \right)e^{{ - i\left( {\left( {k_{p} \left( {\omega + \Omega } \right) - k_{p} \left( \omega \right)} \right)/cos\left( \gamma \right) - k_{THz} \left( \Omega \right)} \right)z}} d\Omega \\ & \;\; - i\frac{{\omega \chi_{eff}^{\left( 2 \right)} }}{{2cn_{p} \left( \omega \right)}}\mathop \smallint \limits_{0}^{\infty } A_{p} \left( {\omega - \Omega ,z} \right)A_{THz} \left( {\Omega ,z} \right)e^{{ - i\left( {\left( {k_{p} \left( {\omega - \Omega } \right) - k_{p} \left( \omega \right)} \right)/cos\left( \gamma \right) + k_{THz} \left( \Omega \right)} \right)z}} d\Omega \\ & \;\; - i\frac{{\omega d_{eff} }}{{cn_{p} \left( \omega \right)cos\left( \gamma \right)}}\mathop \smallint \limits_{0}^{\infty } A_{SH} \left( {\omega + \omega^{\prime},z} \right)A_{p}^{*} \left( {\omega^{\prime},z} \right)e^{{ - i\left( {k_{p} \left( {\omega + \omega^{\prime}} \right) - k_{p} \left( \omega \right) - k_{SH} \left( {\omega^{\prime}} \right)} \right)z/cos\left( \gamma \right)}} d\omega^{\prime} \\ & \;\; - F\left\{ {i\frac{{\varepsilon_{0} \omega_{0} n_{p} \left( {\omega_{0} } \right)n_{2} }}{2}\left| {A_{p} \left( {t,z} \right)} \right|^{2} A_{p} \left( {t,z} \right)} \right\} \\ \end{aligned}$$

$$\frac{{dA_{SH} \left( {\omega^{\prime},z} \right)}}{dz} = - i\frac{{\omega^{\prime}d_{eff} }}{{2cn_{SH} \left( {\omega^{\prime}} \right)\cos \left( \gamma \right)}}\mathop \smallint \limits_{0}^{\infty } A_{p} \left( {\omega ,z} \right)A_{p} \left( {\omega^{\prime} - \omega ,z} \right)e^{{ - i\left( {k_{p} \left( \omega \right) + k_{p} \left( {\omega^{\prime} - \omega } \right) - k_{SH} \left( {\omega ^{\prime}} \right)} \right)z/cos\left( \gamma \right)}} d\omega$$

    Here, $A_{THz} \left( {\Omega ,z} \right)$, $A_{p} \left( {\omega ,z} \right)$, and $A_{SH} \left( {\omega^{\prime},z} \right)$ are the spectral electric field envelopes of the THz, the pump, and the second harmonic of the pump, respectively. These envelopes are defined by $E_{j} \left( {\omega ,z} \right) = A_{j} e^{{ - ik_{j} \left( \omega \right)z}}$, where $j = THz,p,SH,$ and $k_{j} \left( \omega \right)$ -s are the respective wave numbers. The variables $\Omega$, $\omega$, and $\omega^{\prime}$ correspond to the angular frequencies in the THz, pump, and second harmonic spectral ranges, respectively.

In the first equation, $\alpha \left( \Omega \right)$ represents the THz absorption coefficient of GaAs. The effective nonlinear susceptibility in the THz range is denoted by $\chi_{eff}^{\left( 2 \right)} = \frac{4}{\sqrt 3 }86.5\;\;{\text{pm/V}}$, which is the mean of the measurement results of Chang et al.^38^. Additionally, *c* is the speed of the light, and $n_{THz} \left( \Omega \right)$ is the refractive index in the THz range. The two terms on the right-hand side of the first differential equation take into account the linear absorption of the THz pulse and the THz generation by optical rectification of the pump pulse, respectively.

In the second differential equation, $n_{p} \left( \omega \right)$ is the refractive index at $\omega$ frequency, $\gamma = 22.5^\circ$ is the tilting angle of the pump pulse, $\varepsilon_{0}$ is the vacuum dielectric constant, $\omega_{0}$ is the central angular frequency of the pump, $d_{eff} = \frac{2}{\sqrt 3 }83$ pm/V is the effective nonlinear coefficient in the infrared range^39^, and $n_{2} = 5.9 \times 10^{ - 5} \;\;\frac{{{\text{cm}}^{{2}} }}{{{\text{GW}}}}$ is the nonlinear refractive index of the GaAs at 10.6 µm wavelength. The value of the nonlinear refractive index was determined based on the dispersion theory^40^, where the reference nonlinear refractive index at a wavelength of 2 µm is $1.25 \times 10^{ - 4} \;\;\frac{{{\text{cm}}^{{2}} }}{{{\text{GW}}}}$^41^. The individual terms on the right-hand side of the second differential equation describe the cascaded up- and down-conversion – caused by the THz pulse –, the SHG, and the SFM effects.

In the last differential equation, $n_{SH} \left( {\omega^{\prime}} \right)$ is the refractive index at $\omega^{\prime}$ frequency. This differential equation describes the SHG of the pump pulse. It is important to note that our model takes into account the effect of the SH radiation on the pump pulse, and with it, indirectly on the THz generation, too, however for the sake of simplicity, it does not take into account the optical rectification of the SH pulse. The latter expectedly is about 5–10 times smaller than the first one.

**Figure 2:**
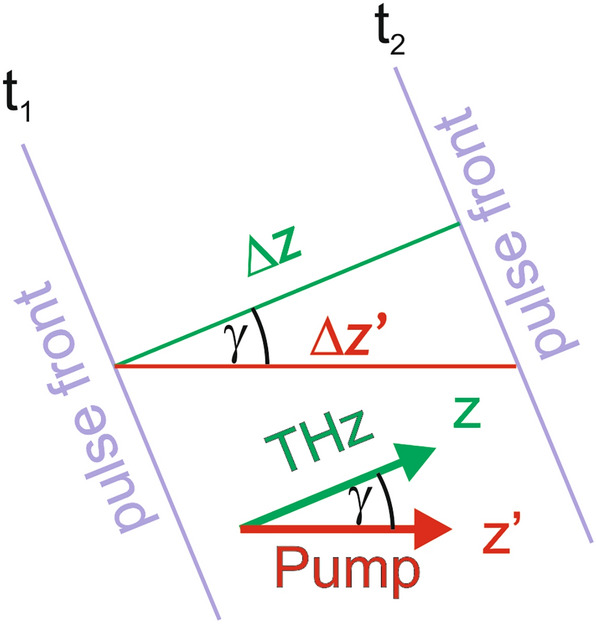
Schematic draw about the coordinate system basis of the calculation and the propagation lengths of the individual pulses (pump (z′, Δz′), THz (z, Δz), the second harmonic(z′, Δz′)) during the same time duration.

To understand $cos\left( \gamma \right)$ factors in the equations, see Fig. 2, where the purple lines represent the pump pulse front at two different time moments. Remember that the pump properties are the same along the pulse front at a given time. The pump pulse propagates in the z′ direction, while the THz pulse propagates in the *z* direction. Therefore, while the THz pulse propagates $\Delta z$ length in the crystal, the pump pulse propagates $\Delta z^{\prime}$ length. The relation between these lengths is given by $\Delta z^{\prime} = \Delta z/cos\left( \gamma \right)$. Hence, this factor must be considered when calculating the phase mismatch between the pump and THz and between the pump and the second-harmonic waves. Additionally, $dz^{\prime} = dz/cos\left( \gamma \right)$ is resulted in $1/cos\left( \gamma \right)$ in the part of the differential equation that describes the second harmonic generation.

Except for the SHG, the nonlinear processes are described in the same way as was discussed in Ref.^11^, and the phase parts were calculated in the same way:

$$k_{p,SH} \left( \omega \right)z = \frac{1}{cos\left( \gamma \right)}\left( {\frac{n\left( \omega \right)\omega }{c} - \frac{{\left( {\omega - \omega_{0} } \right)^{2} }}{2}\frac{{n_{g}^{2} \left( {\omega_{0} } \right)}}{{\omega_{0} cn\left( {\omega_{0} } \right)}}tan^{2} \left( \gamma \right)} \right)z$$

$$k_{THz} \left( \Omega \right)z = \frac{n\left( \Omega \right)\Omega }{c}z$$

## Results and discussion

**Figure 3:**
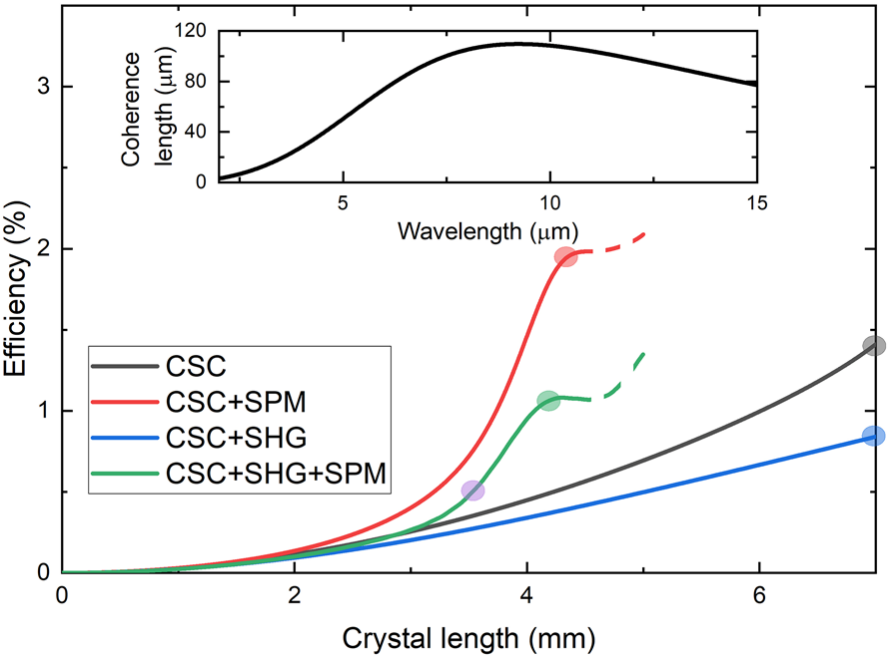
THz generation efficiency as a function of the crystal length for considering four different combinations of processes: The black curve shows the calculation result when the OR (THz generation), the CSC, the absorption of the THz pulses, and the dispersion of both the pump and the generated THz pulses were taken into account. In the case of the blue curve, besides these, the (SHG) second harmonic generation of the pump is also considered. The green and red curves show the result when the self-phase modulation of the pump pulse was also considered, for the green with and for red without taking into account the SHG. The temporal shapes and spectra of the THz pulse belonging to the highlighted points of the efficiency curves are shown in Fig. 4. The inset shows the coherence length of SHG as a function of the fundamental (pump) wavelength.

Figure 3 shows the dependence of THz generation efficiency on the GaAs crystal length for taking into account different processes. Comparison of the different curves provides an opportunity to investigate the significance of the individual physical processes. These calculations assumed pump pulses with 80 GW/cm^2^ intensity and 2 ps pulse duration.

The black curve in Fig. 3 shows the result of the calculation considering only the OR, the cascaded up- and down-conversion (CSC), and the absorption of the THz signal and dispersion of both the pump and THz pulses. In this case, when neither SHG nor SPM were considered, the efficiency shows a square dependence on the crystal length on the whole investigated (up to 7 mm) range. Note that according to the calculations, self-phase modulation can broaden the spectrum of the pump so much that frequency components approach the 0 Hz frequency, causing significant numerical error. In Fig. 3, the range where this numerical error is approached is indicated by the dashed lines.

The coherent length of the SHG at 10.6 µm pump wavelength is one order of magnitude larger than in the near-IR range (see the inset of Fig. 3). Although even the about 100 µm coherence length at 10.6 µm is much shorter than the typical investigated crystal length, yet according to Fig. 3 the presence of SHG process significantly decreases the efficiency of the THz generation (compare the black and blue curves in Fig. 3). For the longest investigated crystal length the efficiency reduces by close to 40% due to the SHG.

According to Fig. 3, the SPM significantly increases (even to above 1%) the THz generation efficiency, both in the presence (green curve) and absence (red curve) of SHG. The reason for this behavior is the combined effect of SPM and dispersion of the pump pulse, resulting in a strong temporal modulation of the pump intensity and an increase of the maximum intensity during the propagation inside the GaAs crystal. (An example of the SPM-caused modulation of the temporal shape of the pump intensity is shown in Fig. 6c).

**Figure 4:**
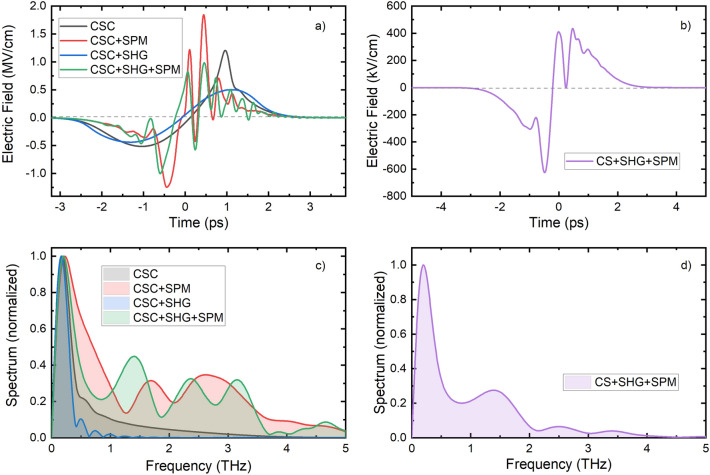
Temporal shapes (**a**) and spectra (**c**) of the generated THz pulses, for taking into account four different combinations of processes, at the crystal length where the efficiency achieves its first maximum (at 7 mm crystal length for CSC and CSC + SHG). The temporal shape (**b**) and the spectrum (**d**) of the THz pulse at crystal length, where the shape is still usable for most types of experiments.

Showing the temporal shapes and spectra of the generated THz pulses at the crystal positions corresponding to maximum THz generation efficiency for considering four different combinations of processes, Fig. 4a and c demonstrate the dramatic effect of SPM for both the THz pulse shape and spectrum. In the absence of SPM, the pulse shape and the spectrum are smooth, irrespective of the presence of SHG. SPM causes a pronounced modulation of both the temporal shape and the spectrum. As mentioned above, the interplay of SPM (resulting in new frequency components of the pump pulse) and the dispersion of the pump pulse results in a strong temporal intensity modulation of the pump pulse. The strong oscillation in the THz pulse shape (and spectrum) results from the modulated pump. (We notice that in Ref.^42^, even an effect of the SPM of the THz pulse was observed, but the investigation of this possibility is behind the scope of this short article.) The strength of SPM depends on the pump intensity and the propagation length (crystal thickness). Decreasing any of them results in a smaller SPM of the pump and a less oscillating THz pulse shape. This effect is illustrated in Fig. 4b and d, showing the THz pulse shape and spectrum for the case of all processes taken into account using the same (80 GW/cm^2^) pump intensity as for Fig. 4a and c, but considering a thinner (3.5 mm, instead of 4.2 mm) GaAs crystal. For this case, the oscillation in the THz pulses shape (Fig. 4b) is less pronounced, and the electric field of the THz signal has only one zero crossing. However, the efficiency drops from 1.1% to 0.5% (see the green and purple dots in Fig. 3).

**Figure 5:**
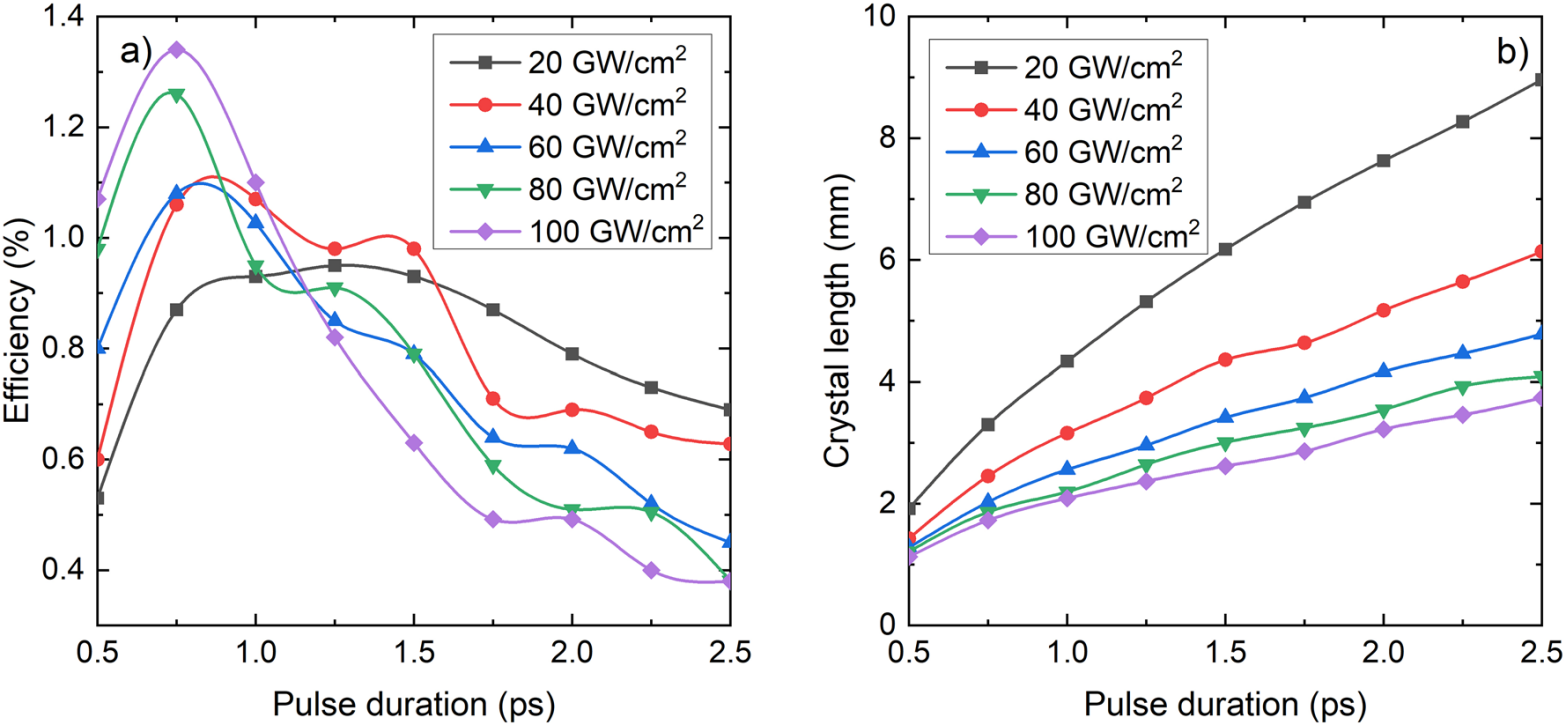
(**a**) THz generation efficiency as the function of the pump pulse duration for 20 GW/cm^2^ (black), 40 GW/cm^2^ (red), 60 GW/cm^2^ (blue), 80 GW/cm^2^ (green), and 100 GW/cm^2^ (purple) pump intensities. The crystal length was chosen for every pump intensity and pulse duration so that the generated THz pulse shape consists of only one zero-crossing. (**b**) The corresponding crystal lengths.

According to Fig. 3, larger than 1% THz generation efficiency can be achieved by ultrashort CO_2_ laser pumped GaAs THz source. However, the generated THz pulse shape is strongly oscillating. Since most of the applications need smoother THz pulse shape, we investigated the efficiency of THz generation for different pulse lengths and pump intensities, choosing the maximum GaAs thickness for which the electric field of the THz signal has only one zero crossing, similar to the shape shown in Fig. 4b. Figure 5a shows the obtained conversion efficiencies, and Fig. 5b shows the appropriate crystal lengths. According to these results, the highest conversion efficiency is 1.3% in the investigated range. This conversion efficiency can be achieved using 0.75 ps long pump pulses with 100 GW/cm^2^ intensity and 1.8 mm long GaAs crystal. About 1.0% efficiency can be achieved if the pulse duration is on the broad 0.75–1.5 ps range. Longer pulse durations belong to smaller optimum pump intensities and longer crystal lengths. Interestingly, for a 1.1 ps pump duration, our model predicts about the same efficiency, independently of the pump intensity. In this case, from the point of view of SPM, the larger intensity probably can be perfectly compensated by the shorter crystal length.

**Figure 6:**
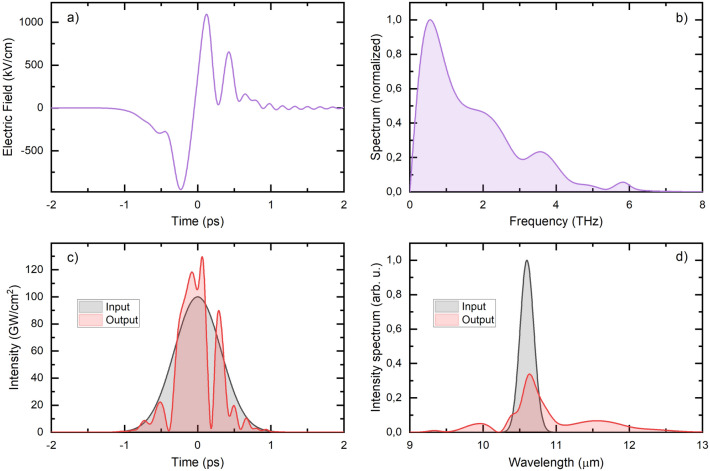
(**a**) Temporal shape of the THz pulse and its (**b**) spectrum supposing pump pulses with 0.75 ps duration, 100 GW/cm^2^ peak intensity, and 1.7 mm GaAs crystal length. (**c**) The initial (black) pump pulse and the pulse shape at the end of the crystal (red) and (**d**) its spectrum in both cases.

Figure 6a and b show the temporal shape and the amplitude spectrum of the THz pulses generated for the parameter set of I_p_ = 100 GW/cm^2^, ∆t_p_ = 0.75 ps, L_GaAs_ = 1.7 mm), resulting in the highest energy conversion efficiency (1.3%). Although the wave shape consists of a weak oscillation, there is only one zero crossing of the electric field, as has been demanded. The spectrum peaks at 0.6 THz and consists of a shoulder and two side peaks with decreasing amplitudes. These later are caused by the small amplitude oscillation in the pulse shape. The reason for this oscillation, as was mentioned above, is the development of a temporal modulation of the pump pulse during its propagation in the crystal. Figure 6c and d display the temporal shape of the pump intensity and the pump spectrum at the GaAs crystal’s entrance (black curve) and exit (red curve) surface, respectively.

The spectrum strongly broadens during the propagation in the GaAs (see Fig. 6d). This is caused mainly by the SPM and the cascading frequency up- and downshifts caused by the feedback of the THz pulse on the pump pulse. The SPM causes a symmetric modulation on the spectrum, while the feedback causes a redshift of the spectrum. Because of these processes and the pump’s dispersion, the pump pulse’s intensity becomes temporally modulated, and a significant increase in the peak intensity occurs. Although it is well known that the dispersion on the THz range can cause oscillation on the trailing part of the generated THz pulse, in this case, the temporal modulation of the pump pulse is probably detrimental.

The peak of the electric field of the THz pulse after exiting the GaAs crystal is as high as 1000 kV/cm.

## Conclusion

The possibility of developing a CO_2_ laser pumped high energy THz source was investigated, for the first time to the best of our knowledge, using an advanced numerical code taking into account both the SPM and (for the first time) the SHG of the pump. Supposing that the well-known GaAs crystal is used for OR of ps—sub-ps CO_2_ laser pulses, the results of the calculation clearly show the essential role of both SPM and SHG. The reason for this is the absence of multiphoton absorption and the long SHG coherence length in the case of long wavelength pumping, respectively. The SPM effect results in spectral broadening and increases the pump intensity during propagation inside the GaAs, in this way fastening the increase of THz generation efficiency with propagation length or initial pump intensity. However, the SPM also causes strong modulation of the pump pulse shape, which results in a distorted THz pulse shape. This strong modulation can be avoided—at the expense of—decreasing efficiency—using shorter crystal lengths and/or smaller pump intensity. The presence of the SHG process also results in an efficiency decrease, which becomes more severe at larger crystal thickness and/or pump intensity.

However, contrary to the negative effect of SPM and SHG, our simulations predict a significant near 0.8% conversion efficiency by using the already available 2 ps duration pump pulses, and the optimal pump pulse length of 0.75 ps results as high as 1.3% conversion efficiency. These values can be expected to be further improved for example by using pre-chirp of the pump pulse^43^. In summary, the CO_2_ laser pumped GaAs THz source can be a high energy and extremely high plug-in efficiency device, which can be used, for example, for THz-driven particle accelerators.

Our conclusions are applicable not only for CO_2_ laser pumping but also for any pump laser or OPA working on the ≈ 4–12 μm wavelength range, where low order multiphoton absorption is not possible, and the SHG coherence length is longer than a few tens of μm.

## Data Availability

Data associated with this research are available and can be obtained by contacting the corresponding author upon reasonable request.

## References

[1] Hebling J, Almási G, Kozma IZ, Kuhl J (2002). Velocity matching by pulse front tilting for large-area THz-pulse generation. Opt. Express.

[2] Hebling J, Yeh K-L, Hoffmann MC, Bartal B, Nelson KA (2008). Generation of high-power terahertz pulses by tilted-pulse-front excitation and their application possibilities. J. Opt. Soc. Am. B.

[3] Zhang B (2021). 1.4-mJ high energy terahertz radiation from lithium niobates. Laser Photon. Rev..

[4] Salén P (2022). Matter manipulation with extreme terahertz light: Progress in the enabling THz technology. Phys. Rep..

[5] Fleischer S, Zhou Y, Field RW, Nelson KA (2011). Molecular orientation and alignment by intense single-cycle THz pulses. Phys. Rev. Lett..

[6] Nanni EA (2015). Terahertz-driven linear electron acceleration. Nat. Commun..

[7] Xu H (2021). Cascaded high-gradient terahertz-driven acceleration of relativistic electron beams. Nat. Photon..

[8] Pálfalvi L, Fülöp JA, Tóth G, Hebling J (2014). Evanescent-wave proton postaccelerator driven by intense THz pulse. Phys. Rev. Spec. Top. Acceler. Beams.

[9] Ravi K, Huang WR, Carbajo S, Wu X, Kärtner F (2014). Limitations to THz generation by optical rectification using tilted pulse fronts. Opt. Express.

[10] Wang L, Kroh T, Matlis NH, Kärtner F (2020). Full 3D + 1 modeling of tilted-pulse-front setups for single-cycle terahertz generation. J. Opt. Soc. Am. B.

[11] Tóth G (2021). Performance comparison of lithium-niobate-based extremely high-field single-cycle terahertz sources [Invited]. Chin. Opt. Lett..

[12] Pálfalvi L (2017). Numerical investigation of a scalable setup for efficient terahertz generation using a segmented tilted-pulse-front excitation. Opt. Express.

[13] Tóth G (2019). Numerical investigation of imaging-free terahertz generation setup using segmented tilted-pulse-front excitation. Opt. Express.

[14] Tóth G (2019). Single-cycle scalable terahertz pulse source in reflection geometry. Opt. Express.

[15] Nugraha PS (2019). Demonstration of a tilted-pulse-front pumped plane-parallel slab terahertz source. Opt. Lett..

[16] Krizsán, G. *et al.* In *2021 Conference on Lasers and Electro-Optics Europe and European Quantum Electronics Conference.* cc_3_2 (Optica Publishing Group, 2022).

[17] Hebling J (2008). Generation of high-power terahertz pulses by tilted-pulse-front excitation and their application possibilities. J. Opt. Soc. Am. B.

[18] Blanchard F (2014). Terahertz pulses generation from bulk GaAs by a tilted-pulse-front excitation at 1.8 mm. Appl. Phys. Lett..

[19] Polónyi G (2016). High-energy terahertz pulses from semiconductors pumped beyond the three-photon absorption edge. Opt. Express.

[20] Fülöp JA (2016). Highly efficient scalable monolithic semiconductor terahertz pulse source. Optica.

[21] Mbithi NM (2022). Investigation of terahertz pulse generation in semiconductors pumped at long infrared wavelengths. J. Opt. Soc. Am. B.

[22] Kumar M, Biswas AK, Biswas T, Joshi J, Rana LB, Yadav RK, Kaul R (2019). Maximizing the efficiency of a compact helium-free TEA CO2 laser: Experimental results and theoretical simulation. Opt. Laser Technol..

[23] Polyanskiy MN, Pogorelsky IV, Babzien M, Palmer MA (2020). Demonstration of a 2 ps, 5 TW peak power, long-wave infrared laser based on chirped-pulse amplification with mixed-isotope CO2 amplifiers. OSA Contin..

[24] Panagiotopoulos P, Hastings MG, Kolesik M, Tochitsky S, Moloney JV (2020). Multi-terawatt femtosecond 10 µm laser pulses by self-compression in a CO2 cell. OSA Contin..

[25] Hanna M (2017). Nonlinear temporal compression in multipass cells: Theory. J. Opt. Soc. Am. B.

[26] Kaumanns M (2018). Multipass spectral broadening of 18 mJ pulses compressible from 1.3 ps to 41 fs. Opt. Lett..

[27] Lavenu L (2018). Nonlinear pulse compression based on a gas-filled multipass cell. Opt. Lett..

[28] Krebs N, Probst RA, Riedle E (2010). Sub-20 fs pulses shaped directly in the UV by an acousto-optic programmable dispersive filter. Opt. Express.

[29] Shumakova V (2016). Multi-millijoule few-cycle mid-infrared pulses through nonlinear self-compression in bulk. Nat. Commun..

[30] Beetar JE, Rivas F, Gholam-Mirzaei S, Liu Y, Chini M (2019). Hollow-core fiber compression of a commercial Yb:KGW laser amplifier. J. Opt. Soc. Am. B.

[31] Weber MJ (2002). Handbook of Optical Materials.

[32] Tochitsky SY, Ralph JE, Sung C, Joshi C (2005). Generation of megawatt-power terahertz pulses by noncollinear difference-frequency mixing in GaAs. J. Appl. Phys..

[33] Gattass RR, Mazur E (2008). Femtosecond laser micromachining in transparent materials. Nat. Photon..

[34] Thompson DE, McMuller JD, Anderson DB (1976). Second-harmonic generation in GaAs “stack of plates””using high-power CO2 laser radiation. Appl. Phys. Lett..

[35] Hebling J, Stepanov AG, Almási G, Bartal B, Kuhl J (2004). Tunable THz pulse generation by optical rectification of ultrashort laser pulses with tilted pulse fronts. Appl. Phys. B.

[36] Tóth G, Pálfalvi L, Tibai Z, Tokodi L, Fülöp JA, Márton Z, Almási G, Hebling J (2019). Single-cycle scalable terahertz pulse source in reflection geometry. Opt. Express.

[37] Fülöp JA, Pálfalvi L, Almási G, Hebling J (2010). Design of high-energy terahertz sources based on optical rectification. Opt. Express.

[38] Chang TY, VanTran N, Patel CKN (1968). Absolute measurement of second order nonlinear coefficient for optical generation of millimeter wave difference frequencies in GaAs. Appl. Phys. Lett..

[39] Roberts DA (1992). Simplified characterization of uniaxial and biaxial nonlinear optical crystals: A plea for standardization of nomenclature and conventions. IWWW J. Quant. Electr..

[40] Sheik-Bahae M, Hutchings DC, Hagan DJ, Stryland EWV (1991). Dispersion of bound electron nonlinear refraction in solids. IEEE J. Quant. Electron..

[41] Hurlbut WC, Lee Y-S, Vodopyanov KL, Kuo PS, Fejer MM (2007). Multiphoton absorption and nonlinear refraction of GaAs in the mid-infrared. Opt. Lett..

[42] Hebling, J., Hoffmann, M. C., Yeh, K. L., Toth, G. & Nelson, K. A. Nonlinear lattice response observed through terahertz SPM. In *Ultrafast Phenomena XVI: Proceeding of the 16th International Conference Heidelberg, Deutschland* 651–653 (2009).

[43] Bakunov MI, Bodrov SB (2014). Terahertz generation with tilted-front laser pulses in a contact-grating scheme. J. Opt. Soc. Am. B.

